# Venous Thromboembolism Risk in Transgender Women on Feminizing Hormone Therapy: A Narrative Review of Formulation-Specific Risks, Management Strategies, and Evidence Gaps

**DOI:** 10.7759/cureus.111121

**Published:** 2026-06-18

**Authors:** Sai Sushrutha Mudupula Vemula, Niket Shah, Lalitsiri Atti, Maxwell Akanbi, Borys Hrinczenko

**Affiliations:** 1 Internal Medicine, Gandhi Medical College and Hospital, Secunderabad, IND; 2 Internal Medicine, University of Michigan Health-Sparrow, Lansing, USA; 3 Internal Medicine, Michigan State University College of Human Medicine, Okemos, USA; 4 Internal Medicine, Michigan State University, Lansing, USA; 5 Hematology and Medical Oncology, McLaren Greater Lansing, Lansing, USA; 6 Oncology, Michigan State University, Lansing, USA; 7 Oncology, McLaren Greater Lansing, Lansing, USA

**Keywords:** anticoagulation, estrogen, gender-affirming hormone therapy, transgender females, venous thromboembolism (vte)

## Abstract

The transgender population is growing, with more people seeking gender-affirming care. While estrogen-based gender-affirming hormone therapy (E-GAHT) is essential for transfeminine individuals, most evidence on associated venous thromboembolism (VTE) risks is outdated and no longer used in clinical practice. Routine primary anticoagulant prophylaxis is not recommended for low-risk individuals initiating E-GAHT. In selected patients with prior VTE, known thrombophilia, or multiple major thrombotic risk factors, prophylactic anticoagulation may be considered on an individualized basis after assessment of bleeding risk and shared decision-making, although this approach is not currently supported by high-quality prospective evidence. Contemporary 17β-estradiol regimens show substantially lower VTE rates, with transdermal formulations showing the most favorable safety profile. In the perioperative setting, continuation of E-GAHT or a reduced dose in high-risk groups with standard VTE prophylaxis, guided by Caprini scoring, is recommended, as no increase in postoperative VTE was observed in multiple retrospective cohorts and meta-analyses. In the transgender population, discontinuation of GAHT following a VTE event can cause significant emotional and psychological distress. Recent studies support continuing GAHT, preferably at a reduced dose, while initiating therapeutic anticoagulation, with extended or indefinite duration considered through shared decision-making.

## Introduction and background

Most combined oral contraceptive (COC) pills contain ethinyl estradiol (EE), a potent synthetic estrogen used with progestins to suppress ovulation, whereas estrogen-based gender-affirming hormone therapy (E-GAHT) uses bioidentical 17β-estradiol at physiologic doses, often alongside testosterone suppressants, to mimic premenopausal hormone levels. A meta-analysis of 15 observational studies in postmenopausal women showed that oral estrogen significantly increased the risk of venous thromboembolism (VTE) (RR 1.63, 95% CI 1.40-1.90), deep vein thrombosis (DVT), and possibly stroke compared to transdermal estrogen [1]. High-dose EE (100 µg/day) combined with cyproterone acetate carried a 45-fold increased VTE risk (6.3% incidence) and is no longer used in feminizing hormone therapy given its significantly higher thrombotic and cardiovascular risk [2,3].

COCs use EE at supraphysiologic doses of up to 50 mcg/day to suppress ovulation and regulate menstruation and carry the highest VTE risk; EE is therefore avoided in both menopausal and feminizing hormone therapy due to its potent hepatic procoagulant effects, including greater activated protein C (APC) resistance and larger reductions in protein S and antithrombin compared to 17β-estradiol [4,5]. Menopausal hormone replacement therapy (HRT) uses lower-dose bioidentical 17β-estradiol, including oral (0.25-1 mg/day), transdermal (14-100 mcg/day), or topical gel (~1 mg/day), to restore physiologic estrogen levels and alleviate vasomotor and urogenital symptoms, with intermediate VTE risk driven primarily by oral formulations. In contrast, feminizing hormone therapy uses bioidentical 17β-estradiol delivered orally (2-6 mg/day), transdermally (0.1-0.4 mg twice weekly), or by injection (estradiol valerate 5-20 mg every two weeks or estradiol cypionate 2-10 mg weekly), targeting serum levels of 100-200 pg/mL to induce and maintain secondary female characteristics while minimizing supraphysiologic estrogen exposure, per Endocrine Society and Boston University transgender care guidelines, and carries the lowest thrombotic risk, particularly with transdermal delivery [4]. This article was previously presented as a meeting abstract at the 67th Annual American Society of Hematology (ASH) Meeting on December 6, 2025.

**Figure 1:**
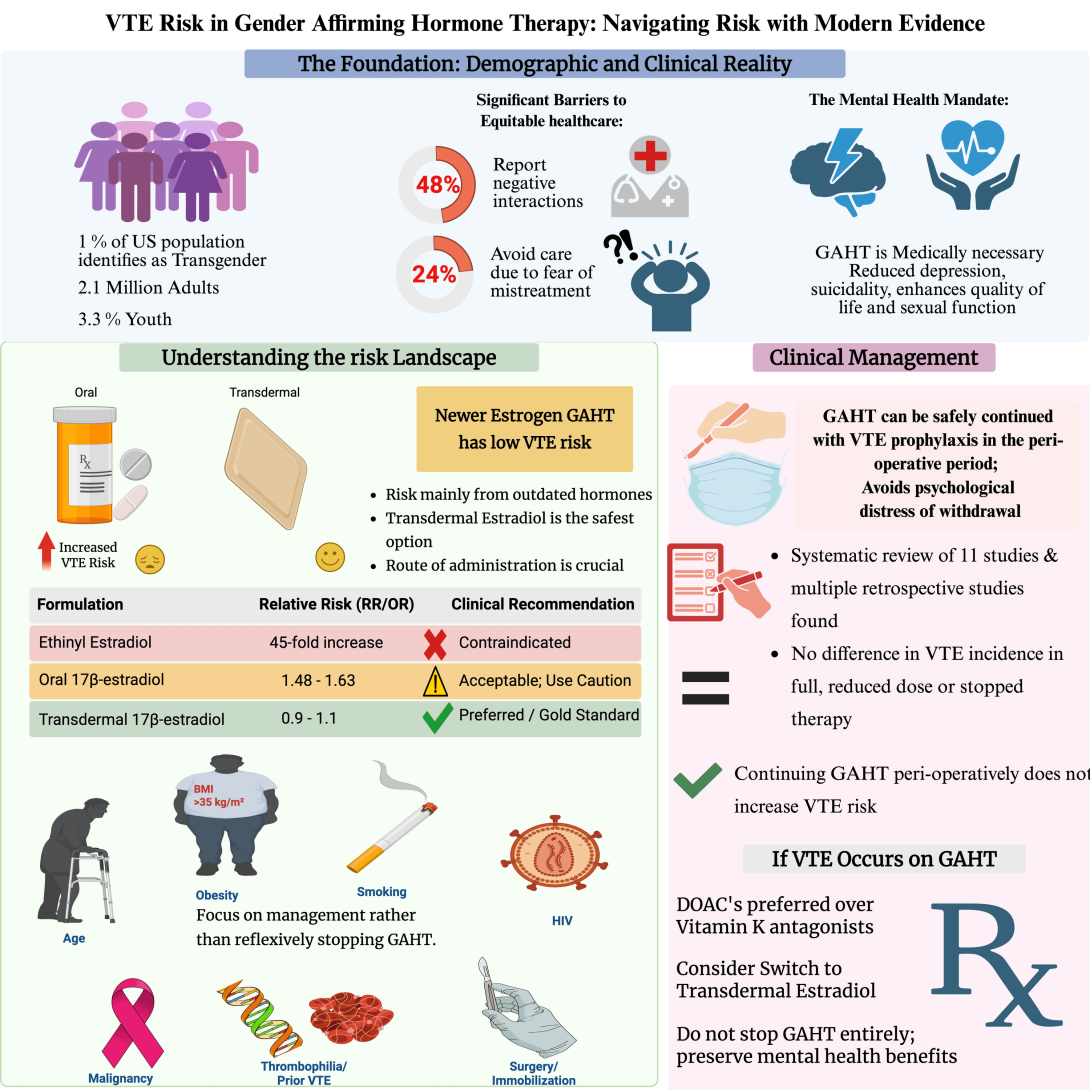
Visual abstract summarizing venous thromboembolism (VTE) risk stratification by estrogen formulation, modifiable and non-modifiable risk factors, perioperative management strategies, and the clinical approach to VTE occurring during estrogen-based gender-affirming hormone therapy (E-GAHT) in transgender women. Figure 1 was created using BioRender (BioRender.com). No AI image generation tools were used in the creation of this figure.

VTE, encompassing DVT and pulmonary embolism, is among the most clinically relevant risks of E-GAHT, yet robust transgender-specific trial data remain limited, and direct extrapolation from cisgender menopausal or contraceptive studies is limited by differences in dosing, antiandrogen use, and baseline risk profiles. This review synthesizes current evidence on VTE risk, formulation-specific considerations, and practical guidance on risk stratification, prophylaxis, and perioperative management in transgender women and highlights gaps requiring further research, as summarized in Figure 1.

## Review

Methods

A narrative literature review was conducted through February 2026 across PubMed, the Cochrane Library, and Google Scholar using the following keywords and their combinations: transgender females, transgender populations, HRT, gender-affirming hormone therapy (GAHT), estrogen, and VTE. The search was restricted to English-language publications, and duplicate studies were excluded. No formal PRISMA protocol was applied, as this was a narrative rather than a systematic review.

Two reviewers independently screened titles and abstracts for relevance, with full-text review performed for potentially eligible studies, and conflicts were resolved by consensus. Studies were included if they reported VTE outcomes in transgender women receiving estrogen-based therapy or provided mechanistically relevant data from cisgender hormone therapy populations. Studies not available in English, those lacking extractable outcome data, and duplicate publications were excluded. Priority was given to transgender-specific cohort studies, systematic reviews, meta-analyses, and clinical guidelines, with cisgender menopausal and contraceptive literature used only where mechanistically relevant. Case series were incorporated to supplement areas with sparse prospective data. No formal risk-of-bias assessment was performed given the narrative design; however, higher-quality evidence, including randomized controlled trials, meta-analyses, and guideline-level recommendations, was weighted more heavily than observational or descriptive studies in drawing conclusions. As with all narrative reviews, full replicability of the search and selection process is inherently limited, and findings should be interpreted within this methodological context.

Prevalence/epidemiology of transgender identity

Recent national estimates from the Williams Institute in 2025 indicate that over 2.8 million individuals aged 13 years and older in the United States identify as transgender, representing approximately 1.0% of this age group. This includes 2.1 million adults, representing 0.8% of adults, and a higher prevalence of 3.3% among youth aged 13-17 years. Among transgender adults, 32.7% identify as transgender women, 34.2% as transgender men, and 33.1% as nonbinary adults [6].

Racial and Ethnic Disparities

The racial/ethnic distribution of transgender adults and youth appears similar to that of the broader U.S. population, with minor, nonsignificant variations by ethnicity and geographic prevalence ranging from 0.7% in the South to 0.9% in the Northeast. State-level estimates range from 0.4% in New Mexico to 1.2% in Minnesota, according to Williams Institute estimates [6].

Social and Health Care Disparities

Transgender and gender-diverse populations represent a medically vulnerable group that faces substantial barriers to equitable, high-quality health care. More than 48,000 patients underwent gender-affirming surgery between 2016 and 2020, and many others received E-GAHT, ideally supported by a multidisciplinary care team including endocrinology, hematology, and gynecology, among others [7].

Transgender patients face substantial barriers to medical care, as highlighted by the 2022 USTS survey of 92,329 respondents, which reported high rates of violence, discrimination, economic hardship, and inadequate health care. Insurance-related negative experiences affected 26% of insured respondents, 47% experienced at least one negative interaction with a health care provider, and 24% avoided needed care due to fear of mistreatment. These stressors contribute to disproportionate health disparities, including markedly higher suicide attempt rates, a higher HIV prevalence, increased substance use, and higher cardiovascular risk [8-10].

VTE risk in transgender women on E-GAHT: evidence synthesis

A prevalence meta-analysis of 18 studies including 11,542 transgender women on estrogen therapy found a pooled VTE prevalence of 2% (95% CI: 1-3%), with significant heterogeneity across studies. Meta-regression showed that older age and longer duration of estrogen use were significantly associated with higher VTE risk. In participants aged 37.5 years or older, the prevalence increased to 3%, whereas in younger individuals, the risk was negligible (0%). Shorter estrogen exposure (<53 months) was associated with a lower risk than longer therapy durations [11].

Another retrospective study showed that transfeminine individuals had a higher incidence of VTE, with 2- and 8-year risk differences of 4.1 (95% CI, 1.6-6.7) and 16.7 (95% CI, 6.4-27.5) per 1000 compared to cisgender men, and 3.4 (95% CI, 1.1-5.6) and 13.7 (95% CI, 4.1-22.7) per 1000 compared to cisgender women, with a similar incidence of ischemic stroke and myocardial infarction [12]. A systematic review and meta-analysis showed that the pooled incidence of VTE among transgender women receiving estrogen therapy was 2.3 per 1000 person-years (95% CI, 0.8-6.9), but with significant heterogeneity [13]. Another single-center study, including 40 transgender women receiving subcutaneous estradiol, reported no thromboembolic or metabolic adverse events, supporting the safety and efficacy of subcutaneous estradiol in gender-affirming therapy [14]. A systematic review and meta-analysis across 21 studies showed that transgender women on E-GAHT had a significantly increased VTE risk compared to cisgender men (OR 2.23; 95% CI, 1.93-2.57), while no significant difference was observed between transgender men receiving GAHT and cisgender women [15]. The significant heterogeneity across included studies limits the precision of this pooled estimate and should be considered when interpreting these findings.

Contemporary evidence

In contrast to earlier studies, a retrospective study by Mullins ES et al. (2021) of 611 transgender individuals, in which 28.8% received estrogen and 68.1% received testosterone, found that only five participants required anticoagulation (two for prior thrombosis and three for prophylaxis), and no thrombotic events occurred during therapy. These findings suggest that physiologic, titrated GAHT in adolescents may not significantly increase short-term thrombosis risk [16]. Across multiple retrospective cohorts of more than 7,000 transgender individuals, rates of VTE among those receiving GAHT were lower than prior pooled estimates (VTE ~0.15-0.8%), with no independent association between estrogen exposure and thrombotic risk after adjustment. Although several factors were associated with VTE in univariate analysis, none, including estrogen use, remained significant after adjusting for age, race, and comorbidity burden, implying that observed events were largely driven by age and underlying cardiometabolic or hypercoagulable comorbidities rather than E-GAHT itself [17-19]. In a 2025 large institutional retrospective cohort study of 1,143 transfeminine individuals receiving E-GAHT, the incidence of VTE was low at 1.4%, with most VTE events occurring a median of ~8 years after hormone initiation. The majority of events had associated provoking factors or significant cardiometabolic comorbidities, and unprovoked events were rarely reported [20].

Historical Evidence and Evolution of Formulations

HRT typically uses estradiol (E2) or conjugated equine estrogen (CEE). Early studies of VTE risk in transgender women were conducted using formulations that are now considered obsolete. In a pivotal 1989 retrospective study, Asscheman and colleagues reported a 45-fold increased VTE risk (6.3% incidence) in transgender women receiving high-dose EE (100 µg daily) combined with cyproterone acetate (100 mg daily). This dose was 3-5 times higher than that used in contemporary oral contraceptives (20-35 µg), prompting the discontinuation of EE in gender-affirming care [2]. Another retrospective study by Seal LJ et al. (2012) in transgender women reported lower VTE risk with E2 preparations alone compared to CEE [21]. In another observational study of postmenopausal women using HRT, oral CEE use was associated with a significantly higher risk of VTE (OR 2.08, P = .045) than E2, with a nonsignificant trend toward increased myocardial infarction and no difference in ischemic stroke risk [22]. In a subset of 140 controls, CEE users also demonstrated higher endogenous thrombin potential-based activated protein C resistance (P < .001), reflecting greater procoagulant activity [22].

VTE risk by estrogen formulation

Oral vs. Transdermal

In a meta-analysis by Olié V et al., among postmenopausal women, the pooled risk ratios for VTE were 1.9 (95% CI, 1.3-2.3) among oral estrogen users and 1.0 (95% CI, 0.9-1.1) among transdermal estrogen users, indicating that transdermal estrogen was not associated with increased VTE risk, while oral formulations nearly doubled it; this difference may partly reflect non-equivalent doses and greater hepatic first-pass metabolism [23]. Another meta-analysis including 15 observational studies in postmenopausal women showed that oral estrogen therapy significantly increased the risk of a first episode of VTE (RR 1.63, 95% CI, 1.40-1.90), DVT, and possibly stroke compared to transdermal estrogen therapy, but not myocardial infarction [1]. In another retrospective study of 1,109 transgender individuals, van Kesteren PJ et al. reported that VTE was the primary complication among transgender individuals receiving oral estrogens, but its incidence declined after the adoption of transdermal estradiol in those older than 40 years, with no significant difference in overall mortality compared to the general population [24]. A large observational study using the QResearch and CPRD databases reported ORs of 1.58 (95% CI, 1.52-1.64) for oral estrogen and 0.93 (95% CI, 0.87-1.01) for transdermal estrogen versus no estrogen use [25].

Impact of Progestin Therapy

In transgender women, the use of progestins such as cyproterone acetate has been associated with higher VTE risk. Toorians AW et al. (2003) reported that cyproterone acetate monotherapy increased activated protein C resistance in transgender women, likely due to a prothrombotic effect, suggesting safer alternatives such as spironolactone or gonadotropin-releasing hormone (GnRH) agonists [5]. In an updated meta-analysis of postmenopausal women, oral estrogen significantly increased VTE risk (RR 1.48) compared to transdermal estrogen. Among progestins, micronized progesterone was the safest (RR 0.93, 95% CI, 0.65-1.33), whereas norpregnane derivatives (RR 2.42, 95% CI, 1.84-3.18) and medroxyprogesterone acetate (RR 2.77, 95% CI, 2.33-3.30) carried substantially higher VTE risk [26].

In a large prospective UK cohort of postmenopausal women, combined oral HRT containing medroxyprogesterone acetate carried a significantly higher VTE risk (RR 2.67; 95% CI, 2.25-3.17) compared with other progestins (RR 1.91; 95% CI, 1.69-2.17) [27]; while these findings highlight MPA's comparatively higher thrombotic burden, direct extrapolation to transgender women on E-GAHT warrants caution due to differences in indication, dosing, and co-administered antiandrogen therapy.

Pathogenesis of estrogen-associated thrombosis

Compared to transdermal formulations, oral estrogen induces a marked procoagulant shift that includes increased activated protein C (APC) resistance; elevated levels of factors II, VII, IX, X, XI, XII, fibrinogen, and prothrombin-thrombin fragments; and reduced levels of protein C, protein S, antithrombin, and tissue factor pathway inhibitor (TFPI), likely due in part to hepatic first-pass metabolism. These changes are associated with higher VTE incidence, especially in high-risk groups [23]. Transdermal formulations largely bypass this pathway and show minimal hemostatic changes; therefore, high-risk formulations such as EE and cyproterone acetate should be avoided. Pharmacologic estrogen may also disrupt purinergic signaling and endothelial oxidative homeostasis, further promoting thrombosis through impaired microvascular function and endothelial injury [28]. A meta-analysis demonstrated that VTE risk is highest in the first year of menopausal hormone therapy (OR 4.0, 95% CI: 2.9-5.7), attenuating thereafter but remaining elevated (OR 2.1, 95% CI: 1.3-3.8), with no significant difference observed between unopposed and opposed oral estrogen [29]. A cross-sectional study showed that transgender women on long-term E-GAHT had higher PAI-1 and lower free protein S levels than cisgender men, indicating a mildly prothrombotic profile; however, overall hemostatic parameters, including antithrombin, protein C, PT, and thrombin time, resembled those of cisgender women, suggesting a hormonal shift toward a female pattern [30]. In a prospective study, Scheres LJ et al. (2021) revealed that estrogen-based E-GAHT increased coagulation factors IX and XI and decreased protein C levels, indicating a procoagulant shift that may underlie the elevated VTE risk in transgender women [31].

Risk factors for VTE in transgender women

Well-established risk factors for VTE in the general population include advancing age, trauma/immobilization, malignancy, HIV, obesity, hypertension, diabetes mellitus, tobacco use, hyperlipidemia, combined estrogen-progesterone oral contraceptive therapy, and postmenopausal estrogen therapy [32]. HIV infection is a significant additional VTE risk factor due to HIV-associated hypercoagulability, endothelial dysfunction, malignancy, opportunistic infections, and protease inhibitor therapy. Transgender individuals experience disproportionately higher HIV rates than cisgender peers [33]. Most transgender women who develop VTE have at least one risk factor, such as obesity, tobacco use, clotting disorders, immobilization, or malignancy, among others [32]. A recent meta-analysis showed that increasing age and longer duration of estrogen therapy are associated with elevated VTE risk in transgender women. This contrasts with cisgender postmenopausal and earlier transgender data suggesting that VTE risk may be highest during the first year of estrogen therapy, with lower risk in subsequent years [1,11]. The absence of randomized controlled trials in transgender populations receiving E-GAHT limits the precise assessment of VTE risk factors.

Modifiable Risk Factors

BMI: Higher BMI is associated with increased VTE risk in cisgender individuals, as seen in the LITE study (HR 3.09; 95% CI, 2.26-4.23) for those with BMI >35 kg/m² [34]. Extrapolation from cisgender populations may be inappropriate, as BMI-VTE relationships and estrogen exposure patterns may differ substantially in transgender cohorts. However, there is no high-quality evidence that E-GAHT increases BMI or that elevated BMI correlates with higher VTE risk in transgender individuals, and further studies are needed to assess the long-term impact of E-GAHT on BMI and cardiometabolic outcomes.

Smoking: In a case-control study of cisgender women, current and former smokers had moderately increased VTE risks, with ORs of 1.43 and 1.23, respectively. The OR for VTE was 2.0 in smokers without hormone use, 3.9 in oral contraceptive pill (OCP) users who did not smoke, and 8.8 in those who both smoked and used OCPs compared to nonsmokers without hormonal therapy, though data linking smoking and VTE in E-GAHT remain limited [35].

Non-modifiable Risk Factors

Thrombophilia: Routine thrombophilia screening before E-GAHT is not recommended. Ott J et al. (2010) identified APC resistance in 7.2% and protein C deficiency in 0.4% of 251 transgender individuals receiving GAHT, with no VTE events observed and no difference between transgender women and transgender men (8.0% vs. 5.6%), supporting the feasibility of therapy even in those with thrombophilic abnormalities [36].

Prior VTE: Individuals with known thrombophilia, myeloproliferative neoplasm, or malignancy should receive individualized counseling while receiving E-GAHT. A prior VTE is a strong predictor of recurrence; about 30% experience relapse within 10 years without long-term anticoagulation, necessitating secondary prevention and careful risk-benefit assessment when initiating or continuing E-GAHT [37].

Predictive Biomarkers

Baseline biomarkers may also help predict VTE risk in women taking HRT. In a large case-control study, cisgender individuals with elevated baseline D-dimer levels had a sixfold higher risk of VTE after starting HRT (95% CI, 3.6-9.8) compared with those with normal baseline values [38].

Contraindications to feminizing hormone therapy

Absolute Contraindications

Absolute contraindications to feminizing hormone therapy include end-stage chronic liver disease, active estrogen-sensitive neoplasms, unstable coronary artery disease, and previous thromboembolic disease due to an underlying hypercoagulable state, per Coleman E et al. [7], Michel A et al. (2001) [39], and Hembree WC et al. (2017) [3]. A strong family history of estrogen-sensitive neoplasms should prompt individualized counseling and specialist assessment.

Relative Contraindications and Modifiable Risk Factors

Relative contraindications include cerebrovascular or ischemic heart disease, marked hypercholesterolemia, obesity, severe hypertension, severe liver dysfunction, uncontrolled diabetes, cholelithiasis, and untreated macroprolactinoma. Active smoking, obesity, hypertension, hyperlipidemia, and diabetes should be considered modifiable thrombotic risk factors that require counseling and risk mitigation rather than automatic exclusion from E-GAHT in all cases, and should be optimized before initiating or continuing therapy, as outlined in guidelines by Coleman E et al. [7], Michel A et al. (2001) [39], and Hembree WC et al. (2017) [3].

Thrombophilia screening and primary prophylaxis

Routine thrombophilia screening is not recommended before initiating hormone therapy, as clinical risk factors offer higher predictive value. A recent review recommends prophylactic anticoagulation for transgender patients initiating GAHT who have multiple VTE risk factors, although this has not been formally endorsed by major societies [40]. The UCSF transgender care guidelines advise against routine aspirin prophylaxis or screening for thrombophilic mutations. For transgender women with a personal or family history of VTE or a confirmed hypercoagulable state, anticoagulation should follow standard guidelines, and transdermal estrogen may be initiated after informed discussion. Prophylactic apixaban or rivaroxaban may be considered in selected individuals with prior VTE or multiple risk factors and low bleeding risk. Informed consent should address risks, benefits, alternatives, and modifiable risk factors, emphasizing a risk-reduction approach given the substantial mental health and quality-of-life benefits of E-GAHT [4].

Perioperative management of E-GAHT

E-GAHT in transfeminine individuals is often discontinued in the perioperative period to mitigate the risk of thrombotic complications. However, perioperative cessation of E-GAHT can trigger withdrawal symptoms, including hot flashes, night sweats, sleep disturbances, and worsening gender dysphoria and anxiety, potentially impairing recovery and making discontinuation challenging for high-risk patients.

In a retrospective study of 329 transgender women undergoing gender-affirming vaginoplasty, VTE occurred in only one patient (0.4%) in the group that paused E-GAHT and in none of those who continued, despite a mean age of 41 years and elevated BMI; nearly all received bioidentical estrogen and perioperative enoxaparin prophylaxis [41]. In another retrospective study of transgender women receiving estrogen and undergoing primary vaginoplasty, Kozato A et al. (2021) reported only one VTE event, occurring in the group in which estrogen was suspended preoperatively, with no events among those who continued therapy [42].

A systematic review of 11 studies found very low perioperative VTE rates in transgender individuals undergoing facial feminization surgery, with similar incidence whether E-GAHT was stopped or continued [43]. In another retrospective study including transgender individuals undergoing facial feminization surgery, only one postoperative VTE occurred (0.10%), with no difference in VTE incidence among those who continued full-dose hormones, reduced their dose, or stopped therapy [44].

Available retrospective evidence suggests that continuation of contemporary E-GAHT may be reasonable in selected patients receiving standard perioperative VTE prophylaxis; however, decisions should be individualized according to baseline thrombotic risk, surgical risk, route of estrogen administration, and patient preference.

Risk-Stratified Perioperative Approach

Hospitalized patients are routinely assessed for VTE risk using established tools such as the Caprini and Rogers scores for surgical patients and the Padua, IMPROVE, and Kucher scores for medical patients [45]. Although no VTE risk calculators are specific to transgender individuals, most models do not incorporate sex as a variable and can therefore be applied to transgender patients. The Caprini and Kucher models include estrogen-containing hormone therapy as a risk factor, but this mainly indicates postmenopausal or contraceptive estrogen use rather than E-GAHT. Despite these limitations, the Caprini score remains the most commonly used pragmatic tool for perioperative VTE risk stratification, but it has not been specifically validated in transgender individuals receiving E-GAHT. Therefore, Caprini-based recommendations should be interpreted in conjunction with clinical judgment, the route and dose of estrogen, mobility status, surgical complexity, and patient preferences [40,45].

Management of VTE in patients receiving E-GAHT

Acute Management

Given the limited data in transgender populations, individuals who develop VTE while on E-GAHT should be managed per standard anticoagulation guidelines. Direct oral anticoagulants are favored over vitamin K antagonists and low-molecular-weight heparin for the treatment of VTE because of their lower bleeding risk [46]. Some expert guidelines recommend discontinuing E-GAHT during acute VTE treatment and restarting estrogen via the transdermal route after a risk/benefit discussion with the patient, with at least one case series reporting favorable outcomes after 3 months of a direct oral anticoagulant, followed by transition to transdermal estrogen [47]. Anticoagulation may be discontinued after the acute treatment period if a clear, reversible, provoked cause is identified. For unprovoked VTE, indefinite anticoagulation should be discussed for as long as E-GAHT is continued, after an upfront informed discussion with patients about the risks and benefits, noting that most patients prefer to maintain estrogen but are willing to adjust the dose or route per UCSF guidelines.

Duration of Anticoagulation and Recurrent VTE

In a prospective cohort of cisgender women from the Leiden Thrombophilia Study, VTE recurrence was 9.7 per 1000 patient-years after COC discontinuation, rising to 27.3 per 1000 patient-years with continued or restarted COC use and to 35.1 per 1000 patient-years while actively taking COCs among those whose initial VTE was non-estrogen-related [48]. Although randomized trials of COC resumption after VTE are lacking, a similar randomized controlled trial in postmenopausal women with prior VTE, the EVTET trial, was halted early due to markedly increased recurrence in the HRT group (10.7% vs. 2.3%) [49].

A large study compared VTE recurrence with and without hormonal therapy among 1,888 cisgender women treated with rivaroxaban or enoxaparin/VKA. Recurrent VTE incidence was 3.7% per year while on hormonal therapy and 4.7% per year off therapy (HR 0.56; 95% CI: 0.23-1.39), indicating that continuing estrogen therapy alongside full-dose anticoagulation is generally safe with no increased risk of recurrent VTE [50].

Due to limited transgender-specific data, anticoagulation recommendations are largely extrapolated from cisgender women [40]. One recent expert consensus recommends indefinite anticoagulation for VTE occurring on transdermal estrogen to reduce recurrence risk [51], and bleeding or recurrence risk scores, such as HAS-BLED, HEMORR2HAGES, DASH, and Vienna, may guide shared decision-making. No universal algorithm exists for treatment duration; indefinite anticoagulation is favored by some experts and UCSF guidelines for transgender women who plan to remain on estrogen, with the rationale that anticoagulation will offset any ongoing thrombotic risk from continued estrogen therapy, and management should be individualized to balance thrombotic risk against the psychological impact of hormone withdrawal [40].

Limitations

The absence of RCTs in transgender individuals receiving E-GAHT limits the precise assessment of VTE risk. Key limitations include heterogeneity in study designs, formulations, and follow-up periods; limited data on subcutaneous administration; unknown long-term risks; and the need for transgender-specific VTE risk calculators. The significant heterogeneity across included studies limits the precision of this pooled estimate and should be considered when interpreting these findings. Although some of these findings derive from cisgender menopausal hormone therapy populations, they provide mechanistic and formulation-specific insights that may inform E-GAHT risk assessment. However, direct extrapolation should be made cautiously because estrogen doses, indications, baseline characteristics, and co-administered therapies differ substantially.

Future directions

A qualitative study of 15 U.S. Midwest hematologists reported variable comfort with gender-affirming care due to limited training, with participants highlighting the need for structured education, EMR improvements, and better transgender-specific thrombosis data; one-third had recommended or initiated thromboprophylaxis before E-GAHT [52].

Future research should prioritize large prospective studies with standardized VTE outcomes, direct comparisons of E-GAHT formulations and routes, and development of transgender-specific prediction models and biomarker-based risk stratification. In clinical practice, care should integrate the current evidence with shared decision-making to optimize thrombotic safety while preserving the established mental health and quality-of-life benefits of E-GAHT.

## Conclusions

VTE represents an important but manageable risk in transgender women receiving E-GAHT. Historical estimates of markedly elevated thrombotic risk were largely driven by high-dose EE formulations that are no longer in clinical use and should not be extrapolated to contemporary practice. Current evidence with 17β-estradiol, particularly transdermal formulations, demonstrates substantially lower VTE rates. Clinical management should be individualized through shared decision-making, prioritizing transdermal estrogen in those with identifiable risk factors and avoiding EE and high-dose conjugated estrogens. Routine thrombophilia screening or primary prophylaxis is not indicated in low-risk individuals. Perioperative decision-making should be individualized, as continuation of E-GAHT with standard prophylaxis does not appear to increase the risk of surgical thrombotic complications. Importantly, VTE risk alone should not prompt automatic discontinuation of E-GAHT; rather, risk-reduction strategies and close monitoring should guide clinical decisions while preserving the well-established benefits of gender-affirming care.
